# Endoscopic Biopsy as Quality Assurance for Endoscopic Services

**DOI:** 10.1371/journal.pone.0078557

**Published:** 2013-11-12

**Authors:** King-Wah Chiu, Shue-Shian Chiou

**Affiliations:** Division of Hepato-Gastroenterology, Department of Internal Medicine, Kaohsiung Chang Gung Memorial Hospital, and Chang Gung University, College of Medicine, Taiwan, Republic of China; University Hospital Llandough, United Kingdom

## Abstract

Gastroendoscopy (GS) procedures are not only performed by gastroenterologists (GE) but also by hepatologists (HT) in many countries. Endoscopic biopsy (EBx) remains the gold standard for the investigation and documentation of esophago-gastro-duodenal pathology. EBx is subjectively performed by an endoscopist, and the level of skill and experience of the endoscopist may affect the quality of the endoscopic service. Reasons for this discrepancy included lack of experience practitioners to order EBx when required of GS issues between in GE and HT limit access. Ideally, services should be safe and of high quality. This study assessed the EBx/GS ratio as the endoscopic quality assurance as an index of GS services. This was a cohort study of endoscopists at Kaohsiung Chang Gung Memorial Hospital, a teaching hospital in southern Taiwan. There were 34,570 episodes of EBx in 199,877 GS procedures. The 25 endoscopists were divided into GE (n = 13) and HT (n = 12) groups, and correlation coefficients were calculated over a 14.5-year duration of intervention. The Trimmean of EBx/GS was 19.29% in 14.5 years (34570/199877 with Trimmean 0.2 percentile ratio correlations), and the Pearson correlation coefficient was 0.90229. There were significantly more EBx procedures in the GE group than in the HT group at 1 and 5 years (21.5% vs. 15.1% and 20.9% vs. 17.3%, respectively, P<0.00001). Junior GE attempted significantly more EBx than both the senior GE (24.06% vs. 20.41%, P<0.0001), and junior HT (24.06% vs. 13.2%, P<0.0001). In conclusion, quality assurance for gastrointestinal endoscopy involves numerous aspects of unit management and patient safety. Quality measures used with the EBx/GS ratio may be one of the best ways to ensure the quality of endoscopic procedures in a teaching hospital.

## Introduction

In clinical practice, gastroendoscopy (GS) procedures are performed by both gastroenterologists (GE) and hepatologists (HT) in many countries. An increased demand for endoscopic procedures has led to the provision of these services without satisfactory quality assessment methods [1]. The importance of performing high-quality endoscopic procedures has increased [2], in particular to prevent acquired infections after the procedures [3]–[6]. In GS, endoscopic biopsy (EBx) remains the gold standard for the investigation and documentation of esophageal, gastric and duodenal pathology [7]. EBx is subjective performed by an endoscopist, and the level of skill and experience of the endoscopist may affect the quality of the endoscopic service. Reasons for this discrepancy included lack of experience practitioners to order EBx when required of GS issues between in GE and HT are limited access. Ideally, services should be safe and of high quality. An interesting point of view that focus on the attempt of EBx was investigated the difference between the GE and HT. We proposed this study to try to draw up a guideline along with the endoscopic quality assurance for the adoption of this practice that EBx/GS ratio to be an index for the clinical investigation.

## Methods

According to the database of computerized records from the endoscopic unit of Kaohsiung Chang Gung Memorial Hospital from January 1998 to June 2012, 215,046 gastroendoscopic procedures (including 864 esophagoscopies, 650 naso-gastroscopies and 213,532 gastroscopies) were performed by 27 attending physicians. According to the Digestive Endoscopy Society of Taiwan, all GE and HT are permitted to perform endoscopic services after completing two years of fellowship training in a hepato-gastroenterology program. In our endoscopic unit, GE/HT were defined as those who own more than 50% documents of gastroenterological/hepatological related publications in Pub Med, respectively. The digestive physicians with 10 years or more experience and/or had credit above associated professor were defined as senior attending physicians. The digestive physicians with less than 10 years of experience were defined as junior attending physicians. One physician retired and one left during the study period, and therefore 25 physicians were enrolled in this study (13 GE: 9 senior and 4 junior; 12 HT: 9 senior and 3 junior). In total, 34,570 episodes of EBx and 199,877 GS were analyzed. Because the junior physicians did not have 10 years of experience, 1-year (short-term) and 5-year (mid-term) investigations were used in the statistical analysis. The indications for upper endoscopies for the GE and HT are shown in Table 1. Because there could be more than one indication, the number of indications was higher than the number of patients. No severe complications after the endoscopic biopsies such as bleeding, perforations and infections were noted. A small amount of bleeding is inevitable after an endoscopic biopsy, and all cases stopped spontaneously or by endoscopic hemostasis.

**Table 1:** Indications for upper endoscopies for the gastroenterologists and hepatologists.

Indications for endoscopy*		GE (%)	HT (%)	*P* value
UGI bleeding		2597 (1.1)	911 (0.9)	<0.0001
PU		149 (0.1)	142 (0.1)	0
Esophagus	GERD	7716 (3.4)	5037 (4.9)	<0.0001
	Ulcer	4786 (2.1)	2348 (2.3)	<0.0001
	Cancer	2584 (1.1)	525 (0.5)	<0.0001
	Polyp	1016 (0.4)	278 (0.3)	<0.0001
	Submucosa tumor	1421 (0.6)	258 (0.3)	<0.0001
	Varices	10523 (4.6)	6686 (6.6)	<0.0001
	Mallory Weiss	188 (0.1)	12 (0.0)	<0.0001
	Hiatus herniation	5907 (2.6)	1488 (1.5)	0
	Achalasia	403 (0.2)	30 (0.0)	<0.0001
	Barrett	991 (0.4)	158 (0.2)	<0.0001
	Other	5933 (2.6)	488 (0.5)	0
Stomach	Gastritis	57055 (24.9)	21822 (21.4)	0
	Erosion	24982 (10.9)	17613 (17.3)	<0.0001
	Ulcer	30477 (13.3)	14571 (14.3)	0
	Cancer	1610 (0.7)	708 (0.7)	<0.0001
	Polyp	6441 (2.8)	3041 (3.0)	<0.0001
	Submucosa tumor	4719 (2.1)	1021 (1.0)	0
	Lymphoma	312 (0.1)	64 (0.1)	<0.0001
	Chronic Gastritis	9230 (4.0)	1316 (1.3)	0
	Varices	3354 (1.5)	2093 (2.1)	<0.0001
	Angiodysplasia	933 (0.4)	292 (0.3)	<0.0001
	Xanthoma	2058 (0.9)	927 (0.9)	<0.0001
	Marginal ulcer	836 (0.4)	420 (0.4)	<0.0001
	Stomal gastritis	1246 (0.5)	880 (0.9)	<0.0001
	PHG	976 (0.4)	1462 (1.4)	<0.0001
	Endoscopic treatment	2002 (0.9)	909 (0.9)	<0.0001
	EVL	1295 (0.6)	909 (0.9)	<0.0001
	Other	5068 (2.2)	1534 (1.5)	0
Duodenum	Ulcer	19356 (8.5)	9813 (9.6)	0
	Endoscopic treatment	287 (0.1)	80 (0.1)	<0.0001
	Lymphoma	312 (0.1)	64 (0.1)	<0.0001
	Endoscopic treatment	12129 (5.3)	3911 (3.8)	0
**Total**		228892 (100.0)	101811 (100.0)	

* Indications can be more than one selection for each case.

PHG: portal hypertensive gastropathy; PU: peptic ulcer; UGI: upper gastrointestine; EVL: esophageal varices ligation; GE gastroenterologist; GERD; gastroesophageal reflux disease; HT: hepatologist.

### Ethics Statement

All clinical investigations were conducted according to the principles expressed in the Declaration of Helsinki. Written informed consent was obtained from all participants for their information to be stored in the hospital database and used for research. This study was approved by the Institutional Review Board of Chang Gung Memorial Hospital Ethics Committee (No.102-2299B).

### Statistical Analysis

All statistical analyses were performed using the Trimmean method of Microsoft Office Excel 2007 with percent = 0.2. A mean trimmed 20% was computed by discarding the lower and higher 10% of the scores and taking the mean of the remaining scores (http://www.java2s.com/Tutorial/Microsoft-Office-Excel-2007/0420__Statistical-functions/TRIMMEANarraypercentreturnsthemeanoftheinteriorofadataset.htm), and Pearson correlation coefficient analysis was performed (http://www.java2s.com/Tutorial/Microsoft-Office-Excel-2007/0420__Statistical-functions/PEARSONindependentdependentreturnsthePearsonproductmomentcorrelationcoefficient.htm) to create standard quality assurance curves of EBx/GS linear correlation with 95% mean prediction intervals. Comparisons of parameters of the EBx/GS ratio between 1-year and 5-year investigations of the GE and HT were performed using the *X* ^2^ test, Fisher’s exact test, and Student’s *t*-test with SPSS software (version 12.0; SPSS, Chicago, IL, USA). *P* values less than 0.05 were considered to be statistically significant.

## Results

**Figure 1:**
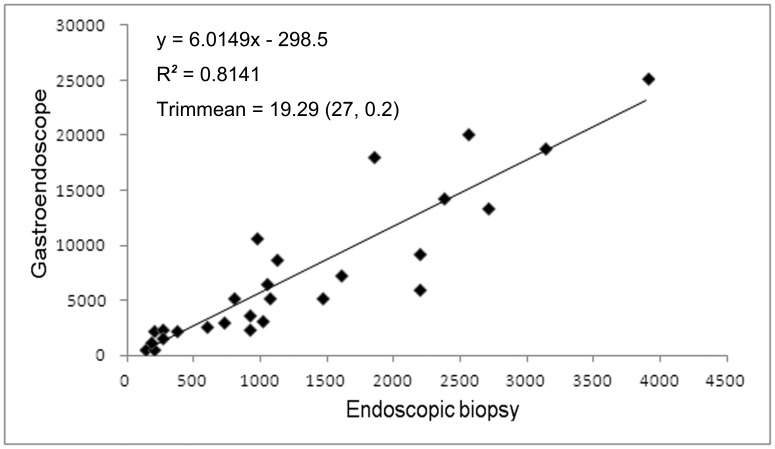
The ratio of the 34,570 endoscopic biopsies and 199,877 gastroendoscopic services with Trimmean (0.2 percent) correction performed by 27 endoscopists including 15 gastroenterologists and 12 hepatologists over 14.5 years.

The ratio of EBx/GS was 17.29% (34570/199877, mean biopsy rate 19.87±8.14%) for the 27 endoscopists over the 14.5-year study period. The Pearson correlation coefficient was 0.90229. The Trimmean of EBx/GS was 19.29% (34570/199877 over 14.5 years) with Trimmean 0.2 percentile ratio correlation, R^2^ = 0.8141 (27, 0.2) (Figure 1) (Table 2). In 1-year and 5-year investigations, the ratio of EBx/GS was significantly higher in the GE group than in the HT group (21.5% vs. 15.1% and 20.9% vs. 17.3%, respectively, P<0.00001)(Table 2). There were no significant differences between the 1-year and 5-year analyses in both the GE and HT groups (Table 2). In the 5-year analysis with the 25 endoscopists, there were significantly more EBx procedures in the GE group than in the HT group (20.9% vs. 17.3%, P<0.0001) (Table 3). The junior GE attempted significantly more EBx procedures than both the senior GE (24.1% vs. 20.4%, P<0.0001) and the junior HT (24.1% vs. 13.2%, P<0.0001) (Table 3). For the HT group, the EBx/GS was significantly higher for the senior than the junior physicians (18.3% vs. 13.2%, P<0.0001) (Table 3). According to the indications for endoscopy (Table 1), the discrepancies between the GE and HT groups were significantly different, especially in esophageal varices, gastric varices and portal hypertensive gastropathy.

**Table 2:** The ratio of endoscopic biopsy/gastroendoscopic procedures and Trimmean modified with Trimmean statistical method of Microsoft Office Excel 2007 between the gastroenterologists and hepatologists.

Category	Gastroenterologist (n = 13)				Hepatologist (n = 12)			
	EBx	GS	Ratio (%)	Trimmean (%)	EBx	GS	Ratio (%)	Trimmean (%)
1 year	2261	10503	21.5^a^	22.23	775	5149	15.1^a^	16.50
5 years	11185	53492	20.9%^b^	22.36	4446	25679	17.3%^b^	16.97
14.5 years*	EBx/GS = 34570/199877, (ratio = 17.29%), (mean = 19.87±8.14%), (Trimmean = 19.29%)							

Statistic analysis used with Trimmean method of Microsoft Office Excel 2007 with percent = 0.2. A mean trimmed 20% was computed by discarding the lowest and highest 10% of the scores and taking the mean of the remaining scores.

* total 27 endoscopists included 15 gastroenterologists and 12 hepatologists; The Pearson correlation coefficient was 0.90229; EBx = endoscopic biopsy; GS = gastroendoscopy;

a, b P<0.0001.

**Table 3:** The 5-year endoscopic biopsy/gastroendoscopy ratio of the 25 endoscopists including 13 gastroenterologists and 12 hepatologists and the distribution of senior and junior physicians in our unit.

		Gastroenterologist				Hepatologist		
		EBx	GS	%		EBx	GS	%
Senior	1	1077	2416	44.6	1	579	2060	28.1
	2	1530	5334	28.7	2	401	1712	23.4
	3	711	2848	25.0	3	406	1791	22.7
	4	965	4140	23.3	4	778	4043	19.2
	5	1170	6120	19.1	5	595	3304	18.0
	6	1318	7452	17.7	6	216	1344	16.1
	7	886	5613	15.8	7	212	1377	15.4
	8	792	5415	14.6	8	415	2859	14.5
	9	973	6825	14.3	9	199	2303	8.6
Junior	10	199	612	32.5	10	253	1600	15.8
	11	717	3017	23.8	11	168	1234	13.6
	12	712	3101	23.0	12	224	2052	10.9
	13	135	599	22.5				
Total		11185	53492	20.9^a^		4446	25679	17.3^a^
All seniors		9422	46163	20.4^b, d^		3801	20793	18.3^b, e^
All juniors		1763	7329	24.1^c, d^		645	4886	13.2^c, e^

EBx: endoscopic biopsy; GS: gastroendoscopy;

a, b, c, d, e *P*<0.0001.

## Discussion

**Figure 2:**
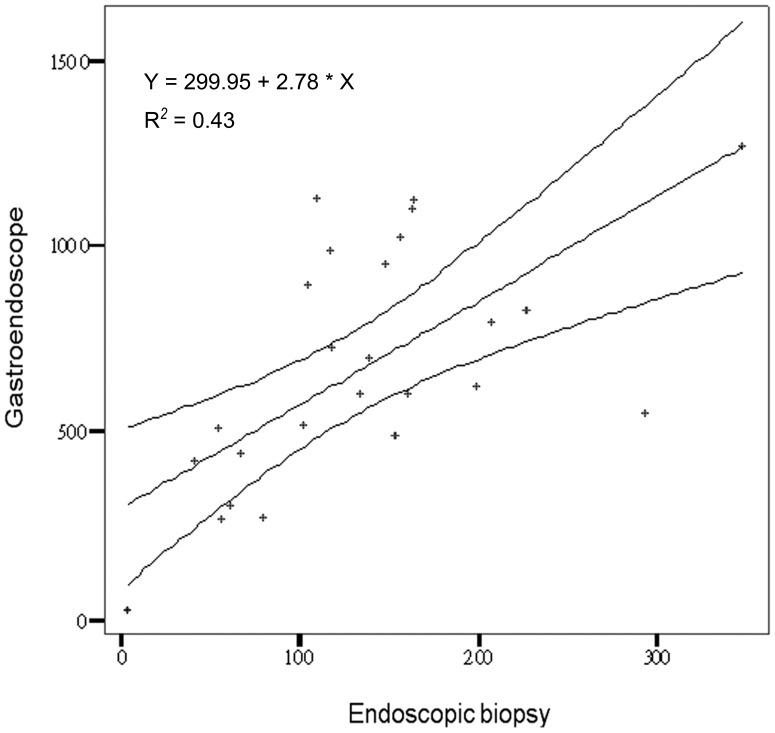
The 5-year ratio of endoscopic biopsies and gastroendoscopic services with 95% mean prediction interval to create a standard quality assurance curve with ±5% border distribution.

Kaohsiung Chang Gung Memorial Hospital is a medical center with 2,715 beds and over 6,900 outpatients and 370 emergency patients. To deliver a high quality medical service, the Hospital adopts a patient-centered approach, safety practices, and encourages innovations in teaching, research and medical services. In this large academic medical cohort study, the average biopsy rate was 17.3% of about 200 thousand endoscopic procedures over a 14.5-year period. Because of differences in EBx for each endoscopist, an appropriate statistical method was used to represent the accuracy, and showed a statistical average of the EBx/GS of 19.87±8.24% with a confidence correlation coefficient of 0.90229. According to the Pearson correlation coefficient, the rate of EBx was statistically related to the GS service. For draw up a standard assurance in endoscopic procedural practice, Trimmean modified with Trimmean statistical method was used to discard the variant data for some endoscopists. A recent study showed that more attending physicians (42%) than fellows (40%) felt that writing a manuscript and belonging to a gastrointestinal society improved knowledge, however the fellows expressed that they needed more practice [8]. In the current study, the higher biopsy ratio for the junior GE (24%) than the senior GE showed that pathological findings can improve experience. That is, the junior GE needed more pathological diagnoses to contribute to the endoscopic findings. In contrast, the lower biopsy ratio for the junior HT (13.2%) than the senior HT (18.3%) suggests a lack of subjective clinical alertness to perform a biopsy, and that the clinical experience of senior HT in interpreting endoscopic findings may not be sufficient. With regards to the indications for the endoscopic procedures, the causes were similar in the two groups. It should be noted that the hepatologists may have been looking for varices and therefore less likely to be taking biopsies in patients with dyspepsia. The large variation in EBx/GS ranging from 8.64% to 44.58% makes it difficult to calculate a confident mean. A mean trimmed by 20% is computed by discarding the lower and higher 10% of the scores and taking the mean of the remaining scores. The Trimmean (27, 0.2) of this study was 19.29%, and the R^2^ value was 0.8141, showing that a 19.3% EBx rate was a quality assurance reference for daily clinical GS services. This is a simple method to calculate a reference mean from a large endoscopic unit for endoscopic assurance of quality. There was no evidence to suggest that a higher EBx/GS mean (44.58%) from the senior GE contributed to better results for the early detection of cancer than the HT or junior GE. If there is no apparent reason for this discrepancy, it is a so-called aberration of waste medical resources. Stand on the physician education that it may be an evidence of the deviant behavior with medical waste. Educating physicians is an essential step in establishing a broader culture of compliance and improved integrity in a healthcare system, extending beyond Medicare and Medicaid [9]–[10]. Therefore, this behavior needs to be corrected [11]. In contrast, a lower EBx/GS mean down to 8.64%, the endoscopic unit staff should have in charge to make a sense or alarm to correct or review the malpractice for the patient safely in a large academic teaching hospital because of the risky for missing diagnosis. How to measure the assurance of the endoscopic service is very important [12]–[14]. Therefore, the value of the present study is in suggesting the mean EBx/GS as a guideline to cover both GE and HT in the clinical endoscopic service. We also attempted to calculate an acceptable range in 95% mean prediction interval with ±5% distribution for the clinical reference as a standard quality assurance curve. The R^2^ was equal to 0.43 (Figure 2). Our results suggest that a ratio of EBx/GS of 19.3±5% (range 14.3% to 24.3%) should be followed by not only GE but also HT in the quality investigation of endoscopic services. There was no significant difference in mean EBx/GS between the 1-year and 5-year observations. Annual performance evaluations should take advantage of this method objectively to observe the physician’s medical performance. This emphasizes the importance of focusing service improvement on enhancing the quality of a patient’s experience of endoscopy and describes the processes used here for quality assurance of endoscopy units [15]. In Taiwan, both GE and HT endoscopists are board certified in gastroenterology. According to the rules of the Digestive Endoscopy Society of Taiwan, all GE and HT are permitted to perform endoscopic service clinically after completing two years of fellowship training in a hepato-gastroenterology program followed by active endoscopic practice and certification from the Digestive Endoscopy Society of Taiwan. There were no definitions for senior or junior certification and no clear definitions to clarify the seniors who were certified in advanced endoscopic techniques. In addition, there were no differences in the endoscopic training and certification between HT and GE. It should be emphasized that biopsy attempts were dependent on the endoscopist. Too many biopsies are a waste of medical resources, and too few biopsies risk misdiagnosis. Therefore, an average curve for clinical reference for each endoscopist may be useful. Bias was present in this study in that misdiagnoses such as discrepancies between macroscopic and microscopic pathological findings was clinical evidence. However, the final diagnosis depended on the pathology. It is very difficult to clarify the misdiagnoses because the endoscopist did not perform a biopsy in each case. Finally, the patient’s satisfaction post-investigation is an important part of the endoscopic service [16], and this will be investigated in future studies. Although it is really difficult (if not impossible) to draw a conclusion only from the EBx/GS ration on the endoscopic quality, it may be one of the usable method to educate by positive measures or open to criticism the assurance of the endoscopic service. In contrast, the data regarding complication after biopsy some like bleeding, perforations, and infections that it is a statement of a negative list approach to medical negligence, it does not focus of this thesis statement nor the endoscopists would like to have. One thing would like to emphasize here, a miss-diagnosis should happen when the lesion needs to be biopsy for pathological confirmation but the endoscopist could not do so. The patients-satisfaction post-investigational also a useful index for the endoscopic service but it is a subjective investigation with questionnaire study because of recall bias [1] when compared with the EBx/GS ration.

In conclusion, quality assurance for gastrointestinal endoscopy involves numerous aspects of unit management and patient safety. Quality measures used with the EBx/GS ratio may be one of the best ways to ensure the quality of endoscopic procedures in a teaching hospital. The acceptable mean of EBx/GS was 19.3% ranging from 14.3% to 24.3% in 1-year evaluations.
